# Test for Insane Persons

**Published:** 1896-07

**Authors:** 


TEST FOR INSANE PERSONS.—Buston Ward, the celebrated English physician, says : There is one infallible symptom indicating whether a person is sane or not. Let a person speak ever so rationally and act ever so sedately, if his or her thumbs remain inactive there is no doubt of his or her insanity. Lunatics seldom make use of their thumbs when writing, drawing, or saluting.

